# Aging in Place Since the COVID-19 Pandemic Onset: A Qualitative Study of Neighborhood Engagement

**DOI:** 10.1093/geroni/igab046.1209

**Published:** 2021-12-17

**Authors:** Lindsay Kobayashi, Melissa Cannon, Gabriella Meltzer, Jessica Finlay

**Affiliations:** 1 University of Michigan, Ann Arbor, Michigan, United States; 2 Western Oregon University, Independence, Oregon, United States; 3 Department of Social and Behavioral Sciences, New York University, New York, United States

## Abstract

The COVID-19 pandemic may fundamentally change neighborhood environments and ways of aging in place. This research aimed to investigate perceptions of and engagement in neighborhoods since the pandemic onset among online survey respondents of the COVID-19 Coping Study. We analyzed a random stratified sample of 500 open-ended responses collected July-September 2020 with quotas for age, gender, race/ethnicity, and education to match the US population aged 55+. Qualitative thematic analysis identified both increased and decreased local activity and varying levels of social interaction, support, and civic engagement. Community characteristics including age structure, socioeconomic diversity, density, housing patterns, weather, and social infrastructure were related to neighborhood perceptions. These interacted with personal characteristics such as duration of residence, living arrangements, family closeness, health status, and preferred lifestyle. Results highlight coping strategies among aging adults and their neighbors, sources of individual and community vulnerability, and opportunities to strengthen social infrastructure and resiliency within neighborhoods.

